# Multi-dimensional knowledge of malaria among Nigerian caregivers: implications for insecticide-treated net use by children

**DOI:** 10.1186/s12936-016-1557-2

**Published:** 2016-10-21

**Authors:** Lauretta Ovadje, Jerome Nriagu

**Affiliations:** School of Public Health, University of Michigan, 1415 Washington Heights, Ann Arbor, MI 48109 USA

**Keywords:** Malaria control, Insecticide-treated net, Malaria knowledge, Behaviour change communication, Nigeria, Misperceptions

## Abstract

**Background:**

Poor malaria knowledge can negatively impact malaria control programmes. This study evaluates knowledge distribution in the domains of causation, transmission, vulnerability, symptoms, and treatment of malaria. It assesses the association between a caregiver’s knowledge about malaria and ownership and use of insecticide-treated nets (ITNs) by children.

**Methods:**

Some 1939 caregivers of young children were recruited through a school-based survey in two Nigerian states. A 20-item, multi-dimensional survey instrument was developed and used to rank each caregiver’s knowledge in five dimensions (cause, transmission, vulnerability, symptoms, treatment of malaria). Scores for each domain were used to create an aggregate knowledge score for each caregiver. The outcome measures were ITN ownership, and ITN use the night and week before the study. Regression models were used to evaluate the relationship between caregiver’s knowledge (individual domains and aggregate score) and ownership and use of ITN after controlling for likely confounders.

**Results:**

The main predictor of ITN use was ITN ownership (r = 0.653; p < 0.001); however, ownership only explains 43 % of variance in net use. Total knowledge index for the study population was significantly associated with both ITN ownership (r = 0.122; p = 0.001) and use (r = 0.095; p = 0.014). The spectrum of caregiver’s knowledge of malaria and its causes captured in the various domains was, however, found to be poor. Fifty percent of the respondents knew that malaria is transmitted by female mosquitoes and 65 % still believe that too much exposure to the sun is a risk factor for malaria. Knowledge of populations most vulnerable to malaria (83 %) and knowledge of malaria transmission (32 %) were the domains with the highest and lowest average correct answers.

**Conclusions:**

There is a need to improve ITN coverage in Nigeria as ITN ownership was associated with ITN use. Additionally, treating knowledge as a multi-dimensional phenomenon revealed that a lot of misperceptions about malaria still exist. Distribution of ITNs through the public/private sector may need to be augmented with tailored behavioural change communication to dispel myths and improve the multi-dimensional knowledge of malaria in the local population.

## Background

Malaria may be conceptually simple to eradicate, but operationally complex. In theory, all it takes to stop mosquitoes from transmitting malaria parasites is a physical barrier (insecticide-treated bed nets, ITNs) between the human host and mosquito and a course of pills that costs pennies to reduce the reservoir of infections in human carriers [1, 2]. This notion is the foundational principle for the current global agenda on malaria eradication which has embraced a three-legged, vector-control approach consisting of distribution of long-lasting insecticide nets, indoor residual spraying and community education for people to understand the importance of sleeping under treated mosquito nets [2–4]. Although huge investments of human and financial resources and many randomized control trials have been conducted on the first two legs of the vector control framework, malaria eradication remains an unattainable goal in many African communities [5, 6]. This paper deals with the various elements of malaria knowledge and how these elements may be associated with ITN ownership and use. It explores the possibility that a shortcoming of current malaria prevention programmes may be the inadequate attention to providing individuals, groups and communities with the knowledge that they need to become better partners in managing mosquitoes and associated health risks. Treating malaria knowledge as a multidimensional construct represents a good framework for integrating the three legs of the vector control strategy into sustainable eradication programmes.

In this study, malaria knowledge is regarded as a multidimensional phenomenon determined by the interplay of five factors (or dimensions): cause of the disease; transmission or how the disease is spread; effective prevention strategies; current treatment regimen; and, vulnerability to the disease [7–9]. Each factor can have emotional, cognitive and spiritual dimensions [10]. Intuitively, poor knowledge is rarely due to lack of understanding of one factor but results from many interlocking factors that cluster in people’s experience, beliefs and definitions of malaria risks [8, 9, 11]. Some studies have found positive association between community ‘education’ and increased use of bed nets, other studies reported no association and a few even found negative association [12]. Typically, health departments and other government agencies, hospitals/clinics, schools, family members, peers, church groups, and the media are critical sources of information ‘communicated’ to/with the community during net distribution [13–15]. Coming from so many actors on malaria control, messages may be inconsistent and even confusing [8, 16]. To reinforce control programmes, such messages should aim at increasing people’s knowledge on malaria in ways that influence the decision and behavioural change towards taking some action to reduce malaria risks in a sustainable manner. This study deals specifically with the association between caregiver malaria-related knowledge and ownership and use of ITNs by children. Knowledge content is important in vector control programmes given the cultural differences in beliefs about malaria as a disease and how to prevent it [13, 15, 16].

Nigeria, which has the highest number of childhood deaths from malaria in Africa [6], has used several strategies to increase ITN coverage, including free public sector campaigns as ‘stand alone’ or integrated with other health activities (e.g., immunizations); free public sector routine distributions through antenatal care (ANC) and expanded programme on immunization (EPI) services; and, subsidized and at cost sales through the commercial sector [17]. However, the use of ITNs remains stubbornly low, reported to be under 50 % [6, 18–20]. While many reasons have been suggested for low ITN use, several studies have singled out poor local knowledge on mosquitoes and malaria as a key stumbling block in malaria control [21–27]. Determining the modifiable factors that drive ITN ownership and use is imperative as more and more resources are targeted to scale up intervention programmes so that the goal of reducing the burden of malaria may be attained in malaria-endemic countries.

This study reports a multidimensional survey instrument developed to assess malaria knowledge (in terms of cause, transmission, prevention, symptoms, vulnerability, and treatment) as predictors of ownership and use of ITN in Nigeria. Specifically, the questions being asked are: (1) How correct is the malaria knowledge in the country? (2) Does high correct malaria knowledge predict ownership of ITNs? (3) Does high correct malaria knowledge of caregivers translate into increased use of ITNs to protect children? The results of this study can be used to enhance the effectiveness of educational interventions in current global efforts to eradicate malaria [6].

## Methods

This was a community-based survey of parents where the sampling frame was children attending primary schools. Young children were the population of interest because they are susceptible to malaria and primary schools were a convenient setting to recruit study subjects with low cost and high efficiency. A pre-piloted, self-administered questionnaire was developed for collection of data [12]. The questionnaire was created by adapting questions from previous knowledge, attitude and practice (KAP) studies [25, 28, 29], and a multiple indicator, cluster survey questionnaire [30]. Additional questions of interest were added and a pre-test of the survey instrument conducted for construct validity. Key outcome measures were ownership and use of ITNs for child protection. The ‘use’ questions consisted of: “Did the child sleep under an ITN in preceding night?” (yes/no); and, “How often did the child sleep under an ITN in past week?” (never, one to three times, four to six times and every day). Data on demographic variables (state of residence, age, educational attainment, gender, etc.) were also collected. The questionnaire had both English and Yoruba versions and the caregivers were told to answer the questions in the language that they were most comfortable with.

Four local government areas (LGAs) in each of the states of Lagos and Oyo in southwest Nigeria were selected based on whether they had participated in ITN distribution campaigns [12]. Malaria transmission occurs throughout the year in these areas but becomes more frequent during the rainy season, which is generally between April and November. The majority of malaria cases in these areas has been ascribed to *Plasmodium falciparum* [6] with the predominant malaria mosquito vector being *Anopheles gambiae* [17]. The survey was conducted in July and December 2011 (encompassing both the rainy and dry seasons).

A list of accredited primary schools in the selected LGAs was received from the Ministries of Education in Lagos and Oyo. Fifteen public and 21 private primary schools were chosen for their accessibility. Children in Grades 1 to 3 in each selected school were given a questionnaire to take home to their caregivers. Where a family had more than one child in the target grades, only one of the children was allowed to participate in the study. To achieve a margin of error of 3 % with 95 % confidence interval and assuming 50 % ITN ownership in each state, the final sample size was calculated to be 1200 caregivers/children. There were 2400 questionnaires given out presuming a minimum participation rate of 50 %. Out of the 2400 questionnaires handed out, 1939 were returned (representing a capture rate of 81 %); 47 of these had a lot of missing data and were excluded in the analysis of the knowledge domains.

The project was determined to be exempt from institutional review board (IRB) by the University of Michigan Health Sciences and Behavioral Sciences IRB since participants were not asked to provide any identifying information (no names, addresses, phone numbers, or other contact details). Anonymizing the data collection was a way of ensuring the ‘truthfulness’ of the answers. In both Lagos and Oyo States, permission to involve children in the selected primary schools in the chosen LGAs was obtained from the appropriate ministries.

### Statistical analysis

All data were entered and cleaned using Microsoft Access and analysed using SPSS version 20. Descriptive statistics were computed for all relevant data. Quantitative variables were summarized using mean, standard deviation and range while frequency tables were created for categorical variables. Knowledge indices were calculated as the sum of the scores for individual items in each sub-scale (domain knowledge index) and for all the items as a whole (total knowledge index). Analysis of variance (ANOVA) and multivariate analysis of variance (MANOVA) were conducted.

The malaria knowledge statements were made up of correct and incorrect statements. The answers were recoded so that respondents who agreed or disagreed with an incorrect statement were given a ‘0’ and ‘1’, respectively. Respondents who agreed or disagreed with a correct statement were given a ‘1’ and ‘0’, respectively. ‘Don’t know’ and no answer responses were treated as incorrect and coded as a ‘0’. Knowledge scores (individual domains and aggregate score) were then created according to the following formula:

$$\frac{{{\text{the}}\;{\text{number}}\;{\text{of}}\;{\text{correct}}\;{\text{statements}}\;{\text{for}}\;{\text{each}}\;{\text{respondent}}}}{{{\text{total}}\;{\text{number}}\;{\text{of}}\;{\text{questions}}}} \times 100$$

This was done so that the knowledge score ranged theoretically from 0 to 100 % and reflected percent of knowledge items answered correctly. Hence, higher scores reflected more correct total malaria knowledge. For most of the statistical analysis, each knowledge score was divided into two categories: higher than the mean score and less than or equal to the mean score. Bivariate analyses of the score and sociodemographic variables were conducted using Chi square tests. A p value of 0.05 or less was considered significant.

Binary logistic regression was used to evaluate the association between each malaria knowledge score and ownership of an ITN. Among ITN owners, multinomial logistic regression was used to assess the use of an ITN by children in the sample the week before the survey with respect to their caregiver’s malaria knowledge scores. Caregivers with scores greater than the mean were compared against caregivers with scores lower than or equal to the mean. All binary and multinomial logistic regression models were adjusted for the following variables: state of residence, gender and age of both child and caregiver, educational level and income range of caregiver. Season and state of residence were automatically correlated hence state of residence was controlled for since only one variable could be used in the models.

## Results

The survey instrument was found to have good psychometric properties. The Cronbach’s alpha was 0.725 considered to indicate good internal consistency (‘reliability’) for such instruments [31]. The alpha value if an item was removed ranged from 0.695 to 0.722 indicating that the items were appropriate descriptors for the study. The inter-item covariance as 0.137 (0.017–0.244) confirmed that there was no auto-correlation among the items. ANOVA with Tukey’s test found no significant additivity in the item measures.

**Fig. 1:**
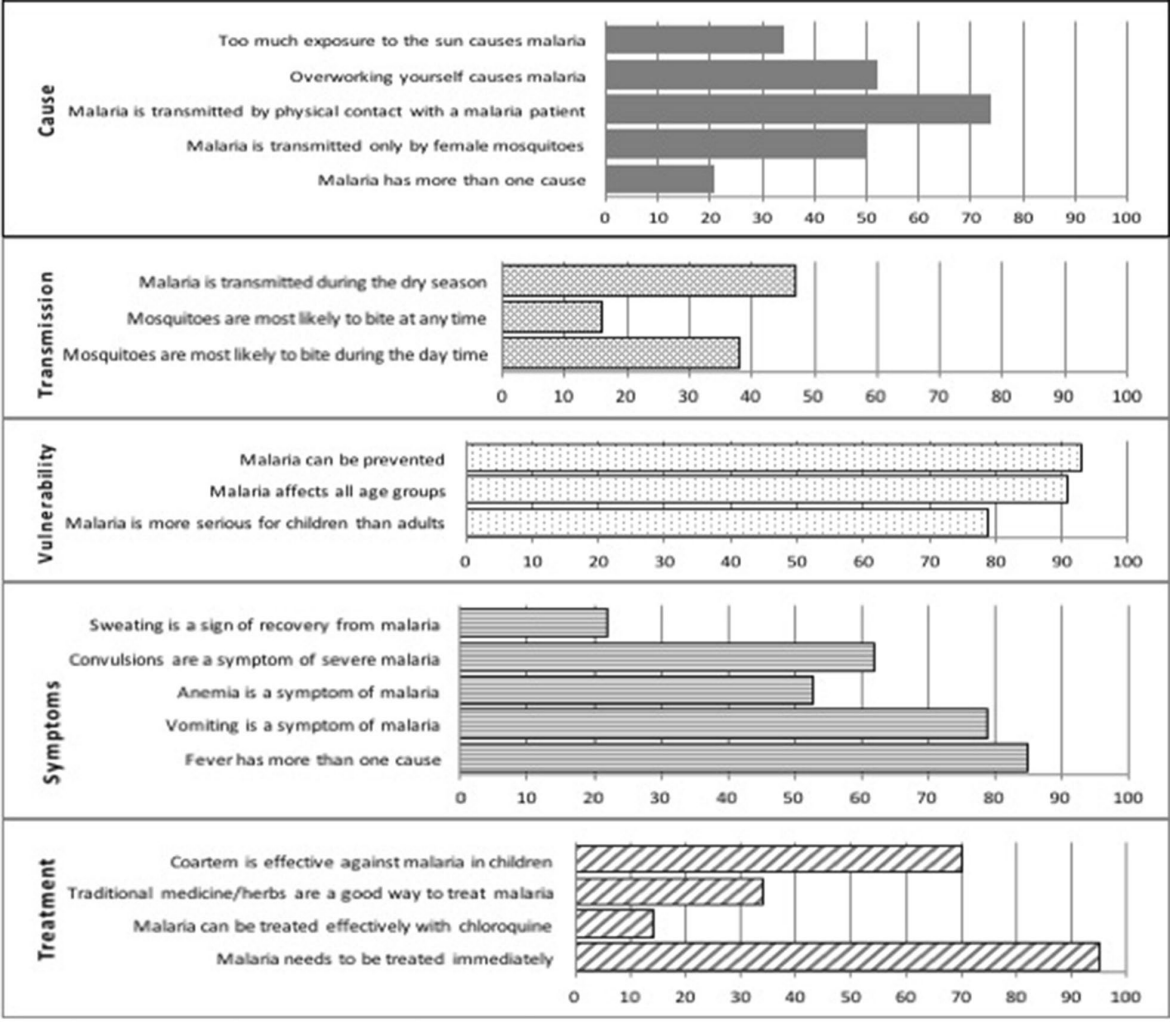
Percentage of correct answers to the malaria knowledge statements

A summary of the sociodemographic characteristics of the respondents is presented in Table 1. The mean correct scores (all questions) for all respondents and ITN owners specifically were 54 and 53 %, respectively (Table 2). Fifty percent of the respondents knew that malaria is transmitted by female mosquitoes. A large proportion still did not know the cause of malaria (Fig. 1). As an illustration, approximately 65 % of caregivers agreed that “*too much exposure to the sun causes malaria*”. Knowledge in the domain of malaria transmission was poor since less than half of the respondents (47 %) knew that malaria could be transmitted during the dry season. The domain with the highest correct answers was Vulnerability. Average correct score of each of the three items in this domain was 80 % or over (Fig. 1). Caregivers’ knowledge about symptoms for malaria was inconsistent with a few participants able to associate anaemia with malaria. While 95 % of the caregivers agreed that malaria needs to be treated immediately, 86 % thought malaria can be treated effectively with chloroquine and 66 % agreed that traditional medicine/herbs are a good way to treat malaria.

**Table 1:** Association between sociodemographic characteristics of the children and caregivers and ITN ownership and use

Variable	ITN ownership			ITN use the week before the survey			Every day	p value
				N	Frequency (%)			
	N	Frequency (%)	p value		Never	Partial		
Location			<0.001					0.09
Lagos	813	474 (58)		460	94 (20)	169 (37)	197 (43)	
Oyo	1126	453 (40)		447	113 (25)	171 (38)	163 (37)	
Gender of caregiver			0.03					0.09
Male	776	345 (45)		341	67 (20)	125 (37)	149 (44)	
Female	1139	565 (50)		550	137 (25)	208 (38)	205 (37)	
Gender of child			0.13					0.32
Male	919	413 (45)		406	83 (20)	154 (38)	169 (42)	
Female	996	483 (49)		471	116 (25)	174 (37)	181 (38)	
Age range of caregiver (years)			<0.001					<0.001
≤30	334	189 (57)		184	22 (12)	89 (48)	73 (40)	
31–40	784	361 (46)		355	91 (26)	127 (36)	137 (38)	
>40	723	312 (43)		305	87 (29)	103 (34)	115 (38)	
Age range of child (years)			0.23					0.59
4–7	1253	570 (46)		561	134 (24)	203 (36)	224 (40)	
8–14	522	254 (49)		247	53 (22)	98 (40)	96 (39)	
Level of education			<0.001					0.04
Primary school or less	345	191 (55)		185	28 (15)	80 (43)	77 (42)	
Secondary school	434	169 (39)		168	41 (24)	57 (34)	70 (42)	
Polytechnic/vocational/technical college	498	212 (43)		206	43 (21)	81 (39)	82 (40)	
University	624	333 (53)		328	92 (28)	117 (36)	119 (36)	
Income range			<0.001					0.14
<20,000 Naira/month	612	262 (43)		259	46 (18)	113 (44)	100 (39)	
20,000 to 100,000 Naira/month	715	342 (48)		334	86 (26)	119 (36)	129 (39)	
>100,000 Naira/month	291	167 (57)		163	40 (25)	62 (38)	61 (37)

**Table 2:** Mean scores for the different malaria knowledge domains

Score	All (n = 1892)	ITN owners (n = 883)
	Mean (SD)	Mean (SD)
Total knowledge	53.77 (14.14)	53.54 (14.76)
Cause	43.86 (28.55)	44.56 (29.85)
Transmission	31.61 (26.39)	31.73 (26.04)
Vulnerability	83.31 (26.74)	80.41 (28.90)
Symptoms	56.93 (25.14)	55.59 (25.81)
Treatment	50.14 (22.16)	49.92 (23.33)

The average total knowledge score (or total knowledge index (TKI)) for the survey instrument was 41.6 ± 6.7, which is slightly higher than half the maximum possible score of 80. The average scores for the sub-scales were 6.8 ± 1.6 for Transmission, 4.9 ± 1.5 for Vulnerability, 9.9 ± 2.5 for Symptoms, and 7.6 ± 1.8 for Treatment. The relationships between ITN ownership, ITN use, TKI, and individual domain scores are shown in Table 3. Regression model of inter-domain associations show that only the Vulnerability/Cause pair was not statistically significant (Table 3).

**Table 3:** Correlations between ITN ownership, ITN use, TKI, and individual domain scores

		Cause	Transmission	Vulnerability	Symptom	Treatment	Ownership of any bed net	Ownership of ITN	Did child sleep under an ITN in preceding night	How often did child sleep under ITN in past
Cause	Correlation coefficient									
	Sig. (2-tailed)									
Transmission	Correlation coefficient	0.271**								
	Sig. (2-tailed)	0								
Vulnerability	Correlation coefficient	0.029	0.145**							
	Sig. (2-tailed)	0.316	0							
Symptom	Correlation coefficient	0.160**	0.184**	0.453**						
	Sig. (2-tailed)	0	0	0						
Treatment	Correlation coefficient	0.139**	0.112**	0.417**	0.400**					
	Sig. (2-tailed)	0	0	0	0					
Ownership of any bed net	Correlation coefficient	0.05	0.01	0.018	0.009	0.05				
	Sig. (2-tailed)	0.07	0.702	0.453	0.773	0.075				
Ownership of ITN	Correlation coefficient	0.092**	0.044	0.052**	0.011	0.078**	0.838**			
	Sig. (2-tailed)	0.001	0.079	0.034	0.722	0.005	0			
Did child sleep under an ITN in preceding night	Correlation coefficient	0.059*	0.023	0.077**	0.021	0.077**	0.557**	0.653**		
	Sig. (2-tailed)	0.034	0.368	0.002	0.487	0.006	0	0		
How often did child sleep under ITN in past	Correlation coefficient	0.068*	0.035	0.078**	0.024	0.064*	0.659**	0.775**	0.861**	
	Sig. (2-tailed)	0.015	0.171	0.002	0.425	0.024	0	0	0	
Total Knowledge Index (TKI)	Correlation coefficient	0.594**	0.501**	0.534**	0.738**	0.612**	0.07	0.122**	0.095*	0.108**
	Sig. (2-tailed)	0	0	0	0	0	0.066	0.001	0.014	0.005

* Correlation is significant at the 0.05 level (2-tailed)

** Correlation is significant at the 0.01 level (2-tailed)

The strongest explanatory factor for the use of ITNs to protect children was ownership of the net (r = 0.653; p < 0.001) and ownership of any net for that matter (r = 0.557; p < 0.001) (Table 3). The TKI was significantly associated with ITN ownership (r = 0.122; p = 0.001) and use (r = 0.095; p = 0.014) but not with ownership of any bed net (r = 0.070; p = 0.66). Each sub-scale was found to be significantly associated with ownership as well as use of the ITN (Table 3). On the other hand, none of the sub-scales was significantly associated with ownership of just any bed net (Table 3). The strongest associations between the sub-scales were between Vulnerability and Symptoms (r = 0.453; p < 0.001), Vulnerability and Treatment (r = 0.417; p < 0.001), and Symptoms and Treatment (r = 0.400; p < 0.001). Although the sub-scales are related (the maximum r^2^ value was 0.205), the associations found in this study suggest that the domains are indeed measuring different elements of knowledge within the study population.

Results from the MANOVA showed that the five knowledge domains were significant influencing factors on both ITN ownership (r = 0.151; p = 0.008) and ITN use (r = 0.166; p = 0.002). However, only a very small fraction of the variance in ITN ownership (2.3 %) and use (2.8 %) could be explained by the knowledge measures. The univariate test results (ANOVAs) for each knowledge domain show that the significant latent variables were related to causes of malaria (β = 0.133, t = 3.22, p = 0.001, r^2^ = 0.032) and malaria prevention (β = 0.075, t = 1.67, p < 0.096, r^2^ = 0.027). The knowledge domain with significant main effects on ITN use were malaria cause (β = 0.121, t = 2.93, p < 0.001, r^2^ = 0.033); malaria prevention (β = 0.138, t = 3.08, p < 0.002, r^2^ = 0.039); and, malaria transmission (β = 0.087, t = 2.15, p < 0.032, r^2^ = 0.030).

Table 4 shows results of the multinomial logistic regression models assessing the relationship between each correct score and ITN use the week before the survey. There was a marginally significant association between higher total correct score on malaria knowledge of a caregiver and child’s use of an ITN every day the week prior to the survey (OR = 1.539; 95 % CI = 0.981–2.413; p = 0.061). A significant association was seen between caregivers’ knowledge of the vulnerable population and child’s partial use of an ITN the week before the survey (OR = 1.863; 95 % CI = 1.178–2.946; p = 0.008). Additionally, higher correct score on malaria symptoms was significantly associated with a higher likelihood of a child using an ITN every day the week before the survey (OR = 1.599; 95 % CI = 1.029–2.486; p = 0.037).

**Table 4:** Association between malaria knowledge scores and ITN use the week before the survey

Score	N	Partial		Everyday	
		OR (95 % CI)	p value	OR (95 % CI)	**p-value**
Total correct score >54 %	347	1.584 (1.002–2.505)	0.49	1.539 (0.981-2.413)	0.061
Total correct score ≤54 %	279	Ref			
Cause >44 %	271	1.091 (0.702–1.696)	0.7	0.883 (0.572–1.364)	0.576
Cause ≤44 %	370	Ref			
Transmission >32 %	444	1.336 (0.841–2.122)	0.219	1.266 (0.799–2.005)	0.316
Transmission ≤32 %	197	Ref			
Vulnerability >80 %	397	1.863 (1.178–2.946)	0.008	1.249 (0.789–1.979)	0.343
Vulnerability ≤80 %	244	Ref			
Symptoms >56 %	395	1.271 (0.811–1.994)	0.296	1.599 (1.029–2.486)	0.037
Symptoms ≤56 %	246	Ref			
Treatment >50 %	498	1.116 (0.633–1.966)	0.705	1.682 (0.978–2.891)	0.06
Treatment ≤50 %	143	Ref			

Multinomial logistic regression models were adjusted for state of residence, income level of caregiver, educational level of caregiver, age, and gender of both child and caregiver

## Discussion

Overall, the multinomial logistic regression showed that the association between high total correct scores and ITN ownership or use was non-significant. However, knowledge in specific domains such as vulnerability and symptoms were significantly associated with ITN use. Additionally, the TKI was significantly associated with ITN ownership and ITN use. Each knowledge domain (cause, transmission, vulnerability, symptoms, and treatment) was associated with ITN ownership and use. Results of the MANOVA showed that the knowledge domains with significant main effects were malaria cause and malaria transmission. The knowledge measures only explained a small fraction of the variance in ITN ownership and use. These results suggest that other factors may be playing a role in ITN ownership and use in this population and highlight the importance of understanding the local context before interventions are conducted. This may explain the discrepancy between the policy assumption that knowledge enhances the ownership and use of ITNs and what is being observed in the field. Some studies have reported no correlation between malaria knowledge and the use of bed nets, including ITNs [21, 22, 32]. By contrast, several studies have found significant associations between measures of malaria knowledge and ITN use [33–36]. The fact that results are contradictory should not be surprising considering that these studies all used different metrics to evaluate malaria knowledge. Additionally, knowledge and behavior are not necessarily directly related; other variables that may contradict (including beliefs, perceptions, economic and household factors) may be responsible for behavior consistent and inconsistent with knowledge [27].

ITN ownership was a determining factor in a child using the ITN in this study. This finding is similar to results from other studies evaluating predictors of ITN use [8, 37]. In this study, 47 % of the study population owned ITNs. The rate for ITN ownership in this study is similar to the 46 % reported in another study following the mass distribution of net campaigns in Lagos State in 2011 [15]. Among ITN owners in this study, 58 % reported having just one net, 25 % had two nets and 10 % had three nets; average number of ITNs owned by participants’ households was 1.7. The mass distribution campaigns allocated two nets per household in Lagos State [38]. In comparison, an average household of participants had 4.2 children and 3.6 adults. Therein lies a major hindrance to ITN use by children, namely, the caregivers did not have enough nets for all the children to sleep under. This lack of access also implies that the ITNs were not being used by vulnerable populations that serve as reservoirs for malaria infection and transmission. Since ITN ownership had a stronger association with ITN use when compared with the total malaria knowledge scores, it is evident that ITN coverage needs to be increased in Nigeria. Studies show that when ITN coverage is >70 %, malaria morbidity and mortality is reduced [39, 40].

However, ITN ownership does not always translate directly to ITN use. Factors such as correct knowledge are particularly important to enable the large proportion of the participants using malaria control strategies to assess the risks and benefits of the methods available to them [12, 32]. Therefore, this study also sought to evaluate correct malaria knowledge in the surveyed states. Surprisingly, misperceptions about the cause, transmission, symptoms, and treatment of malaria still seem to abound.

Knowledge of malaria cause was poor with the average score for correct answers being 44 % (Fig. 1). The score was skewed somewhat by the fact that a majority of caregivers believe that malaria has more than one cause. Cultural influence on this knowledge domain may need to be considered. Attributions of disease causation in Nigerian communities seem to be deeply rooted in cultural beliefs and are multifarious: personal (such as bad habits or negative emotional states); ecologic (e.g., pollution and germs); interpersonal (e.g., actions of others); and, supernatural factors including God, destiny and indigenous beliefs, such as witchcraft or voodoo [41–43]. Even though the score for malaria cause was poor (i.e. less than 50 %), this domain was associated with ITN use as was found in another study from Nigeria [32].

Knowledge of malaria transmission had the lowest correct answers with a mean score of approximately 32 % (Fig. 1). The majority of participants believed that mosquitoes are likely to bite at any time while over 40 % believed that mosquitoes can also bite during the day time. The poor knowledge could be related to the activities of nuisance *Culex* mosquitoes which have a different biting pattern from *Anopheles* mosquitoes that are mostly active from dusk until dawn [44]. This factor has implications for ITN use as it has been suggested that people who believed that they have been exposed to malaria already (i.e., been bitten during the day by mosquitoes) might be less likely to use an ITN at night [45, 46]. Also, less than half of the caregivers knew that malaria is transmitted during the dry season, a potential reason for not using ITNs during the hot and humid summer months. Malaria is endemic in Nigeria and local infection rates may be reduced by low vector density associated with disappearance of ephemeral breeding grounds during the summer [47]. Nevertheless, a substantial risk of malaria transmission exists throughout the year.

Vulnerability to malaria was the domain that had the highest score showing that caregivers are cognizant of the risk of malaria. This should not be surprising since malaria is a common chronic health problem in Nigeria and people generally have a relatively high degree of understanding about its antecedent cause (mosquitoes) as well as know that anyone is at risk of malaria regardless of age. It may also be related to risk communication that stresses the effectiveness and benefits of net-centred vector control methods almost to the exclusion of other malaria control strategies. While results showed that vulnerability was associated with partial use of an ITN, an interesting question is why this domain did not promote behaviour change towards consistent ITN use by children. One suggestion is that this domain is phenomenally linked to local risk perception: a mixed dose of emotion, experience, belief, and knowledge [48], reported to be a predictor for ITN ownership and use [18]. The high infection rate may lead to indifference and contextualization of malaria as normal and unavoidable; in fact, 15 % of the study population self-reported no interest in malaria control. The experience of living in a malaria-endemic area can desensitize one to malaria risks and hence contribute to non-use of bed nets [49].

Knowledge of common malaria symptoms was average (slightly over 50 % of the answers were correct), with the majority of respondents knowing that fever and vomiting are associated with the disease (Fig. 1). The low score to the statement that “sweating is a sign of recovery from malaria” shows good familiarity with fever (antithetical to sweating) as a common symptom of malaria. However, the proportion of respondents who knew that anaemia (53 %) and convulsions (62 %) are symptoms of malaria is less than adequate. Linking malaria to life-threatening complications such as anaemia and convulsions is vital information which can goad a caregiver to use mosquito control measures for protecting children, especially ITNs [27]. In this study, the Symptoms score was not associated with ITN ownership or use (Table 3). However, children are more vulnerable to complicated malaria when compared to adults; therefore, knowledge in this domain may need to be considered in the behavior change communication (BCC) that accompanies mass distribution of nets in Nigeria.

While the majority of participants (over 95 %) knew that malaria needed to be treated immediately, the overall score of correct answers for malaria treatment was 50 %. This finding suggests that most people are still not sure what to use for treatment of childhood malaria in the country. In this study, over 30 % of caregivers believed that traditional herbs/medicine are a good way to treat malaria. Similar findings from northeastern Nigeria and Côte d’Ivoire show that paradoxically, people with sound knowledge of malaria cause and symptoms are still likely to use traditional medicine [50, 51]. Another study in Nigeria found that the use of traditional herbal preparations was a preferred method for malaria treatment with majority of respondents believing that malaria could be prevented with a combination of “Western” medicine and herbal drugs [52]. The efficacy of the herbal medications is basically unknown and some may even contain toxic chemicals, which may complicate the malaria treatment. Compounding the issue of medication is the finding that 30 % of caregivers did not know that Coartem^®^, which is one of the most popular artemisinin-based combination therapy (ACT) drugs being used in Nigeria, is effective against malaria (Fig. 1). This study suggests that the right drugs may not always be used for treating malaria and this can be a contributing factor to drug resistance and continued malaria prevalence.

ACT is recommended by the WHO as first-line anti-malarial treatment and was adopted in Nigeria in 2004 [53]. Based on this policy, ACT drugs are meant to have been in circulation and use for about seven years at the time of this study. It was therefore unexpected to find that a significant proportion of caregivers (86 %) still think that chloroquine is an effective drug for treatment of malaria. Chloroquine was withdrawn in 2005 in Nigeria as first-line malaria treatment because of widespread and high level clinical failure rates across the country [54]. This drug, however, has remained in use because many health care practitioners do not adhere to national and WHO guidelines for treating malaria cases [55, 56]. Also, a large portion of the Nigerian populace gets its drugs from patent medicine vendors (PMVs) who are poorly regulated [57]. Although more than 200 brands of ACT can be bought over the counter in Nigeria [58], study results show a prevailing positive attitude towards malaria treatment with chloroquine. This has implications for the second leg of the tripartite vector control programme in Nigeria.

There are limitations in this study that should be noted. First, the study was based on self-interview and reported ownership and use of ITNs was not validated with actual observation. Second, the cross-sectional nature of this study is limited in its ability to establish a cause and effect relationship between predictors and outcomes. Third, the information collected on ITN use was based on a recall period of the week preceding the survey (i.e., 7 days) so the data could be subject to recall bias and social desirability bias where caregivers might have reported more use by children than their actual use. Lastly, these data are based on children who were actually present in school the day the survey was passed out, hence some of the target children might not have been sampled. Notwithstanding these limitations, this study adds a different perspective to current knowledge about malaria in two Nigerian states and the potential implications for malaria control in the country.

## Conclusions

This study documents a lot of misperceptions about malaria among the Nigerian caregivers surveyed in the two states. Few of the respondents got high scores across all domains of the measurement instrument used, indicating that correct knowledge about malaria is very limited in the two states surveyed. Study data shows that ownership of ITNs was a stronger predictor of ITN use when compared to total knowledge. However, specific knowledge domains were associated with ITN use. Additionally, evidence from other studies shows that ownership does not automatically translate to use. It is definitely important to increase ITN coverage and a lot of resources are currently being invested in trying to achieve this goal especially in malaria endemic countries like Nigeria. However, educating the local populace has also been shown to be important. While efforts to improve ITN coverage occur, local knowledge of different domains of malaria knowledge may need to be measured as an adjunct to efforts to develop tailored and effective educational interventions needed in the current global effort to eradicate malaria. While knowledge is just one of a complex interplay of factors that drive malaria-related behaviours, it affects attitudes towards malaria control and is an important prerequisite for influencing behaviour change.

## Abbreviations

ACT: artemisinin combination therapy
ANC: antenatal care
ANOVA: analysis of variance
BCC: behaviour change communication
EPI: expanded programme on immunization
IRB: institutional review board
ITN: insecticide-treated net
KAP: knowledge, attitudes and practice
LGA: local government area
MANOVA: multivariate analysis of variance
PMV: patent medicine vendor
TKI: total knowledge index
WHO: World Health Organization

## References

[1] malERA Consultative Group on Vector Control (2011). A research agenda for malaria eradication: vector control. PLoS Med..

[2] WHO Global Malaria Programme. Insecticide-treated mosquito nets: A WHO position statement [Internet]. Geneva: World Health Organization; 2007. http://files.givewell.org/files/DWDA 2009/Interventions/Nets/itnspospaperfinal.pdf. Accessed 20 June 2016.

[3] Miller LH, Pierce SK (2009). Perspective on malaria eradication: is eradication possible without modifying the mosquito?. J Infect Dis.

[4] Gates B. We can eradicate malaria-within a generation. https://www.gatesnotes.com/Health/Eradicating-Malaria-in-a-Generation. Accessed 20 June 2016.

[5] Liu J, Modrek S, Gosling RD, Feachem RGA (2013). Malaria eradication: Is it possible? Is it worth it? Should we do it?. Lancet Glob Health..

[6] WHO. World malaria report 2015. Geneva, World Health Organization, 2015. http://apps.who.int/iris/bitstream/10665/200018/1/9789241565158_eng.pdf. Accessed 20 June 2016.

[7] Rhee M, Sissoko M, Perry S, Mcfarland W, Parsonnet J, Doumbo O (2005). Use of insecticide-treated nets (ITNs) following a malaria education intervention in Piron, Mali: a control trial with systematic allocation of households. Malar J..

[8] Birhanu Z, Abebe L, Sudhakar M, Dissanayake G, Yihdego Y, Alemayehu G (2015). Access to and use gaps of insecticide- treated nets among communities in Jimma Zone, southwestern Ethiopia: baseline results from malaria education interventions. BMC Public Health..

[9] Nyunt MH, Aye KM, Kyaw MP, Wai KT, Oo T, Than A (2015). Evaluation of the behaviour change communication and community mobilization activities in Myanmar artemisinin resistance containment zones. Malar J..

[10] Bhuiya A, Mahmood SS, Rana AKMM, Wahed T, Ahmed SM, Chowdhury AMR (2007). A multidimensional approach to measure poverty in rural Bangladesh. J Health Popul Nutr..

[11] Abraido-Lanza AF, Armbrister AN, Florez KR, Aguirre AN (2006). Toward a theory-driven model of acculturation in public health research. Am J Public Health.

[12] Ovadje L (2014). Adherence to the use of insecticide-treated bed nets by Nigerian children.

[13] Iwuafor AA, Egwuatu CC, Nnachi AU, Ita IO, Ogban GI, Akujobi CN (2016). Malaria parasitaemia and the use of insecticide-treated nets (ITNs) for malaria control amongst under-5 year old children in Calabar, Nigeria.. BMC Infect Dis..

[14] García-Basteiro AL, Schwabe C, Aragon C, Baltazar G, Rehman AM, Matias A (2011). Determinants of bed net use in children under five and household bed net ownership on Bioko Island, Equatorial Guinea. Malar J..

[15] Zegers de Beyl C, Koenker H, Acosta A, Onyefunafoa EO, Adegbe E, McCartney-Melstad A (2016). Multi-country comparison of delivery strategies for mass campaigns to achieve universal coverage with insecticide-treated nets: what works best?. Malar J..

[16] Boulay M, Lynch M, Koenker H (2014). Comparing two approaches for estimating the causal effect of behaviour-change communication messages promoting insecticide-treated bed nets: an analysis of the 2010 Zambia malaria indicator survey. Malar J..

[17] Federal Ministry of Health National Malaria Control Programme. Strategic plan 2009-2013; A road map for malaria control in Nigeria. Abuja, Nigeria; 2008. http://www.nationalplanningcycles.org/sites/default/files/country_docs/Nigeria/nigeria_draft_malaria_strategic_plan_2009-2013.pdf. Accessed 20 June 2016.

[18] Ankomah A, Adebayo SB, Arogundade ED, Anyanti J, Nwokolo E, Ladipo O (2012). Determinants of insecticide-treated net ownership and utilization among pregnant women in Nigeria. BMC Public Health..

[19] Idowu OA, Sam-Wobo SO, Oluwole AS, Adediran AS (2011). Awareness, possession and use of insecticide-treated nets for prevention of malaria in children under five in Abeokuta, Nigeria. J Paediatr Child Health..

[20] Ye Y, Patton E, Kilian A, Dovey S, Eckert E (2012). Can universal insecticide-treated net campaigns achieve equity in coverage and use? the case of northern Nigeria. Malar J..

[21] Aikins M, Pickering H, Greenwood BM (1994). Attitudes to malaria, traditional practices and bednets (mosquito nets) as vector control measures: a comparative study in five West African Countries. J Trop Med Hyg..

[22] Agyepong IA, Manderson L (1999). Mosquito avoidance and bed net use in the Greater Accra Region. Ghana. J Biosoc Sci..

[23] Binka FN, Adongo P (1997). Acceptability and use of insecticide impregnated bednets in northern Ghana. Trop Med Int Health..

[24] Okrah J, Traore C, Pale A, Sommerfeld J, Muller O (2002). Community factors associated with malaria prevention by mosquito nets: an exploratory study in rural Burkina Faso. Trop Med Int Health..

[25] Rashed S, Johnson H, Dongier P, Moreau R, Lee C, Crépeau R (1999). Determinants of the permethrin impregnated bednets (PIB) in the Republic of Benin: the role of women in the acquisition and utilization of PIBs. Soc Sci Med.

[26] Winch P, Makemba A, Kamazima S, Lwihula G, Lubega P, Minjas J (1994). Seasonal variation in the perceived risk of malaria: implications for the promotion of insecticide-impregnated bed nets. Soc Sci Med.

[27] Adongo P, Kirkwood B, Kendall C (2005). How local community knowledge about malaria affects insecticide-treated net use in northern Ghana. Trop Med Int Health..

[28] Brieger WR, Onyido AE, Sexton JD, Ezike VI, Breman JG, Ekanem OJ (1996). Monitoring community response to malaria control using insecticide-impregnated bed nets, curtains and residual spray at Nsukka, Nigeria. Health Educ Res..

[29] Deressa W, Ali A (2009). Malaria-related perceptions and practices of women with children under the age of 5 years in rural Ethiopia. BMC Public Health..

[30] National Population Commission (NPC) [Nigeria], National Malaria Control Programme (NMCP) [Nigeria], ICF International. Nigeria Malaria Indicator Survey 2010. Abuja: Nigeria; 2012. https://dhsprogram.com/pubs/pdf/MIS8/MIS8.pdf. Accessed 20 June 2016.

[31] Grau E. Using factor analysis and cronbach’s alpha to ascertain relationships between questions of a dietary behavior questionnaire. Proc Am Stat Assoc. 2007. Section on Survey and Research Methods. Report 600.

[32] Arogundade ED, Adebayo SB, Anyanti J, Nwokolo E, Ladipo O, Ankomah A (2011). Relationship between care-givers’ misconceptions and non-use of ITNs by under-five Nigerian children. Malar J..

[33] Biswas AK, Hutin YJ, Ramakrishnan R, Patra B, Gupte MD (2010). Increased financial accessibility and targeted education messages could increase ownership and use of mosquito nets in Purulia District, West Bengal, India. Trans R Soc Trop Med Hyg.

[34] Bennett A, Smith SJ, Yambasu S, Jambai A, Alemu W, Kabano A (2012). Household possession and use of insecticide-treated mosquito nets in Sierra Leone 6 months after a national mass-distribution campaign. PLoS ONE.

[35] Graves PM, Ngondi JM, Hwang J, Getachew A, Gebre T, Mosher AW (2011). Factors associated with mosquito net use by individuals in households owning nets in Ethiopia. Malar J..

[36] Paulander J, Olsson H, Lemma H, Getachew A, San Sebastian M (2009). Knowledge, attitudes and practice about malaria in rural Tigray, Ethiopia. Glob Health Action..

[37] Ruyange MM, Condo J, Karema C, Binagwaho A, Rukundo A, Muyirukazi Y (2016). Factors associated with the non-use of insecticide-treated nets in Rwandan children. Malar J..

[38] Kilian A, Koenker H, Baba E, Onyefunafoa EO, Selby RA, Lokko K (2013). Universal coverage with insecticide-treated nets—applying the revised indicators for ownership and use to the Nigeria 2010 malaria indicator survey data. Malar J..

[39] Choi HW, Breman JG, Teutsch SM, Liu S, Hightower AW, Sexton JD (1995). The effectiveness of insecticide-impregnated bed nets in reducing cases of malaria infection: a meta-analysis of published results. Am J Trop Med Hyg.

[40] Eisele TP, Lindblade KA, Wannemuehler KA, Gimnig JE, Odhiambo F, Hawley WA (2005). Effect of sustained insecticide-treated bed net use on all-cause child mortality in an area of intense perennial malaria transmission in western Kenya. Am J Trop Med Hyg.

[41] Helman CG (2007). Culture, health and illness.

[42] Vaughn LM, Jacquez F, Baker RC (2009). Cultural health attributions, beliefs, and practices: effects on healthcare and medical education. Open Med Educ. J..

[43] Feyisetan BJ, Asa S, Ebigbola JA (1997). Mothers’ management of childhood diseases in Yorubaland: the influence of cultural beliefs. Health Transit Rev..

[44] Lindsay SW, Snow RW, Broomfield GL, Janneh MS, Wirtz RA, Greenwood BM (1989). Impact of permethrin-treated bednets on malaria transmission by the *Anopheles gambiae* complex in The Gambia. Med Vet Entomol.

[45] Alaii JA, Van Den Borne HW, Kachur SP, Shelley K, Mwenesi H, Vulule JM (2003). Community reactions to the introduction of permethrin-treated bed nets for malaria control during a randomized controlled trial in Western Kenya. Am J Trop Med Hyg.

[46] Kudom AA, Mensah BA (2010). The potential role of the educational system in addressing the effect of inadequate knowledge of mosquitoes on use of insecticide-treated nets in Ghana. Malar J..

[47] Craig M, Snow R, le Sueur D (1999). A climate-based distribution model of malaria transmission in sub-Saharan Africa. Parasitol Today.

[48] Nriagu J, Udofia E, Ekong I, Ebuk G (2016). Health risks associated with oil pollution in the Niger Delta, Nigeria. Int J Environ Res Public Health..

[49] Okwa OO (2003). The status of malaria among pregnant women: a study in Lagos, Nigeria. Afr J Reprod Health..

[50] Essé C, Utzinger J, Tschannen AB, Raso G, Pfeiffer C, Granado S (2008). Social and cultural aspects of “malaria” and its control in central Côte d’Ivoire. Malar J..

[51] Akogun OB, John KK (2005). Illness-related practices for the management of childhood malaria among the Bwatiye people of north-eastern Nigeria. Malar J..

[52] Iriemenam NC, Dosunmu AO, Oyibo WA, Fagbenro-Beyioku AF (2011). Knowledge, attitude, perception of malaria and evaluation of malaria parasitaemia among pregnant women attending antenatal care clinic in metropolitan Lagos, Nigeria. J Vector Borne Dis..

[53] WHO. World malaria report 2008. Geneva: World Health Organization; 2008. http://apps.who.int/iris/bitstream/10665/43939/1/9789241563697_eng.pdf. Accessed 20 June 2016.

[54] Efunshile M, Runsewe-Abiodun T, Ghebremedhin B, König W, König B (2011). Prevalence of the molecular marker of chloroquine resistance (pfcrt 76) in Nigeria 5 years after withdrawal of the drug as first-line antimalarial: a cross-sectional study. South Afr J Child Health..

[55] Meremikwu M, Okomo U, Nwachukwu C, Oyo-Ita A, Eke-Njoku J, Okebe J (2007). Antimalarial drug prescribing practice in private and public health facilities in south–east Nigeria: a descriptive study. Malar J..

[56] Umar MT, Chika A, Jimoh A (2011). Compliance of public health care providers to recommendations of artemisinin based combination therapies in the treatment of uncomplicated malaria in selected PHC centres in Sokoto Northwestern Nigeria. Int J Trop Med..

[57] Nriagu J, Afeiche M, Linder A, Arowolo T, Ana G, Sridhar MKC (2008). Lead poisoning associated with malaria in children of urban areas of Nigeria. Int J Hyg Environ Health.

[58] Palafox B, Patouillard E, Tougher S, Goodman C, Hanson K, Arogundade E (2012). ACTwatch 2009 Supply Chain Survey Results.

